# Supplementary figures and images for: Gut microbiome diversity is associated with sleep physiology in humans
Source: PLoS One. 2019 Oct 7;14(10):e0222394. doi: 10.1371/journal.pone.0222394 (PMC6779243; doi:10.1371/journal.pone.0222394)

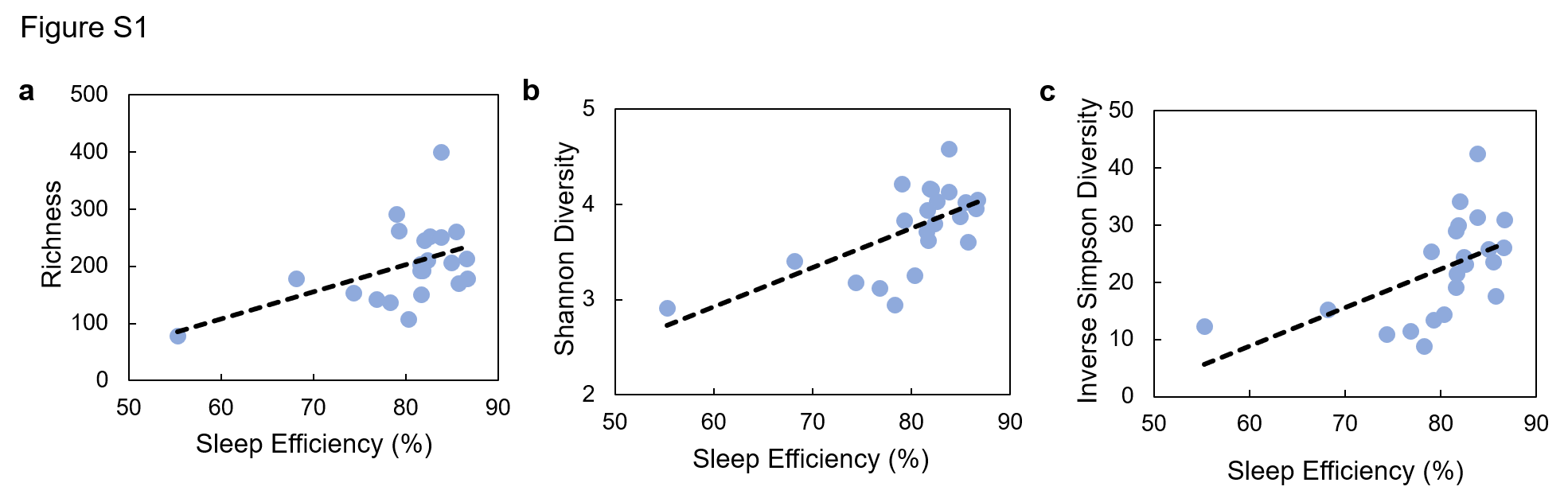

Supplement: S1 Fig — a) Pearson correlation analysis of richness and sleep efficiency (ρ = 0.479, P = 0.001). In all panels, dotted line is a linear line plotted through the data. b) Pearson correlation analysis of Shannon diversity and sleep efficiency (ρ = 0.643, P = 0.001). c) Pearson correlation analysis of inverse Simpson diversity and sleep efficiency (ρ = 0.540, P = 0.009). (PNG) [file pone.0222394.s001.png]

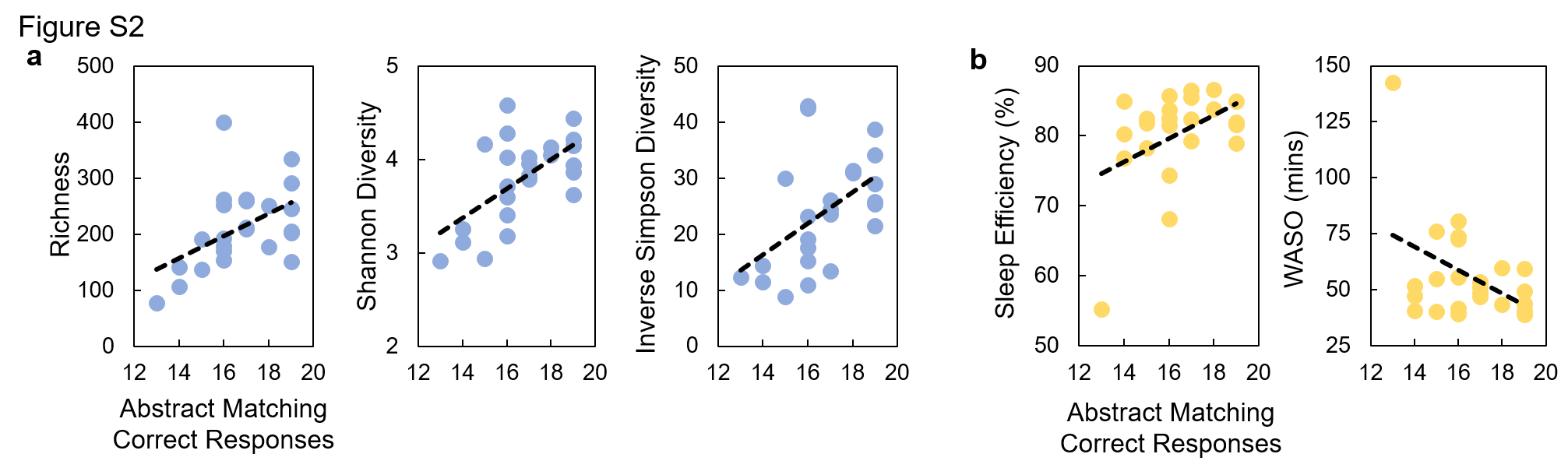

Supplement: S2 Fig — a) Pearson correlation analysis richness (ρ = 0.489, P = 0.015), Shannon diversity (ρ = 0.607, P = 0.002), and inverse Shannon diversity (ρ = 0.501, P = 0.013) with the number of correct abstract matching responses. In all panels, dotted line is a linear line plotted through the data. b) Pearson correlation analysis of sleep efficiency (ρ = 0.405, P = 0.044) and WASO (ρ = -0.424, P = 0.035) with correct abstract matching responses. (PNG) [file pone.0222394.s002.png]

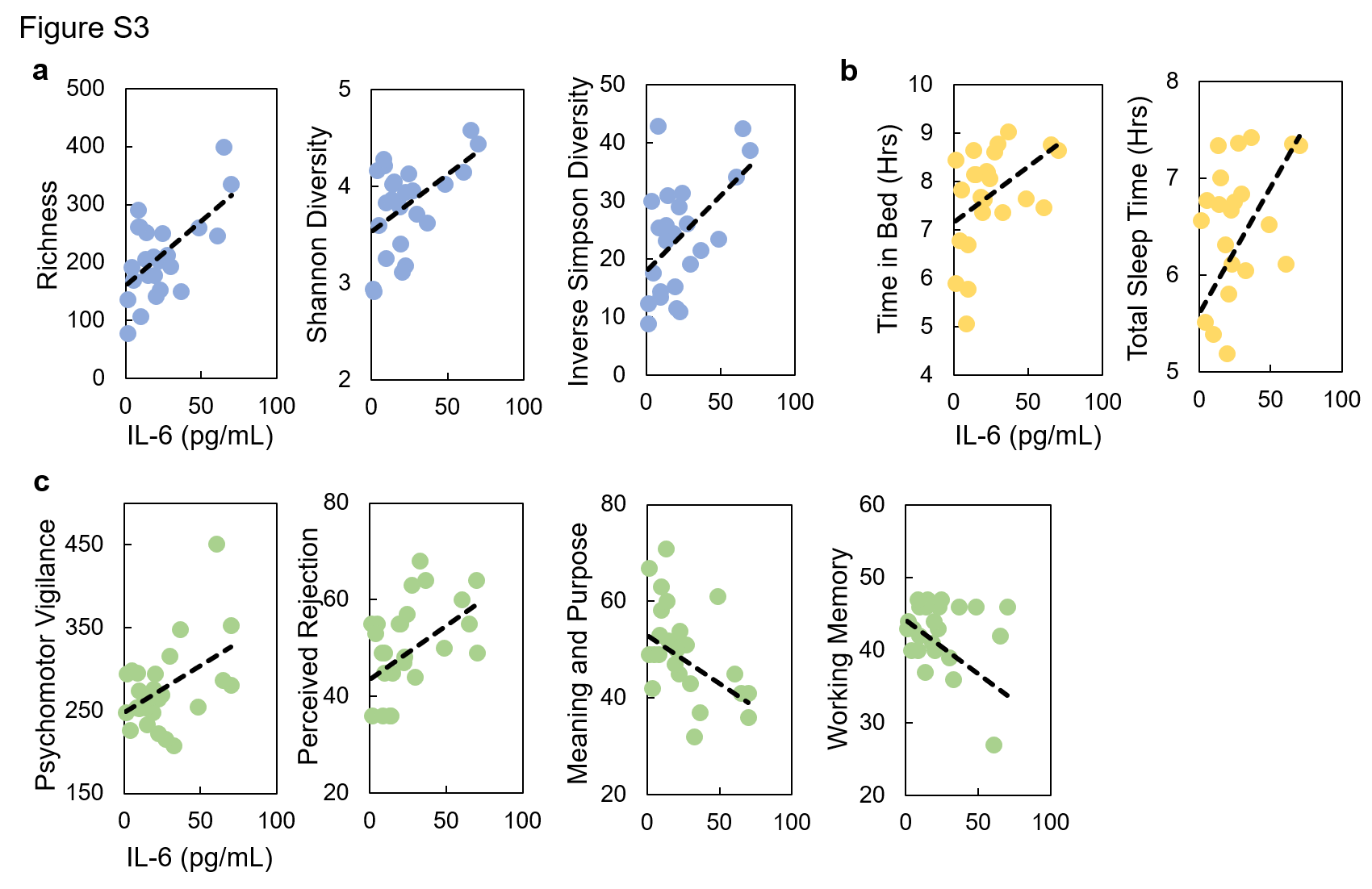

Supplement: S3 Fig — a) Pearson correlation analysis of IL-6 with richness (ρ = 0.612, P = 0.001), Shannon diversity (ρ = 0.508, P = 0.011) and inverse Simpsons diversity (ρ = 0.521, P = 0.009. In all panels, dotted line is a linear line plotted through the data. b) Pearson correlation analysis of IL-6 with time in bed (hrs, ρ = 0.439, P = 0.032) and total sleep time (ρ = 0.476, P = 0.019). c) Pearson correlation analysis of IL-6 with psychomotor vigilance (ρ = 0.469, P = 0.016), perceived rejection (ρ = 0.451, P = 0.024), working memory (ρ = -0.388, P = 0.045), and meaning and purpose (ρ = -0.507, P = 0.010). (PNG) [file pone.0222394.s003.png]

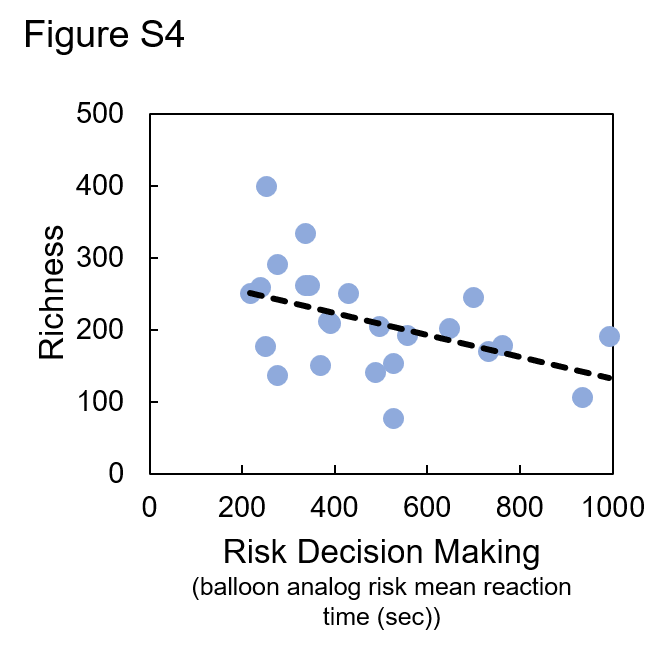

Supplement: S4 Fig — (PNG) [file pone.0222394.s004.png]
