# Supplementary material for: Gut microbiome diversity is associated with sleep physiology in humans
Source: PLoS One. 2019 Oct 7;14(10):e0222394. doi: 10.1371/journal.pone.0222394 (PMC6779243; doi:10.1371/journal.pone.0222394)
Supplement: S1 Table — (DOCX) [file pone.0222394.s005.docx]

| **IL-1β** | **Co-variate** | **Pearson correlation coefficient (ρ)** | **Significance (two-tails, P)** |
| --- | --- | --- | --- |
|  | Richness | -0.128 | 0.550 |
|  | Shannon diversity | -0.176 | 0.410 |
|  | Inverse Simpson diversity | -0.315 | 0.133 |
|  | Time in Bed | -0.073 | 0.735 |
|  | Total sleep time | -0.095 | 0.659 |
|  | Onset latency | 0.077 | 0.719 |
|  | Sleep efficiency | -0.044 | 0.837 |
|  | WASO | 0.006 | 0.980 |
|  | Number of awakenings | 0.039 | .855 |

**Table S2: Correlations coefficients and P values of associations between measures of sleep, IL-6 and cognition/emotion in our interaction network, and bacterial genera.**

| **Phylum** | **Taxa** | **Sleep Efficiency** | | **Total Sleep Time** | | | | **Number of Awakenings** | | **IL-6** | | | | | **Risk Task Mean Reaction Time** | | **Abstract Matching Correct Responses** |
| --- | --- | --- | --- | --- | --- | --- | --- | --- | --- | --- | --- | --- | --- | --- | --- | --- | --- |
| Actinobacteria | *Brevibacterium* |  | |  | | | | -0.44 (0.04) | |  | | | | |  | |  |
|  | *Corynebacterium* |  |  |  |  |  |  | -0.43 (0.04) | |  |  |  |  |  |  |  |  |
|  | *Dermabacter* |  |  |  |  |  |  | -0.41 (0.05) | -0.41 (0.05) |  |  |  |  |  |  |  |  |
| Firmicutes | *Geobacillus* |  |  |  |  |  |  |  | |  |  |  |  |  | 0.5 (0.02) | 0.5 (0.02) |  |
|  | *Leuconostoc* |  |  |  |  |  |  |  |  |  |  |  |  |  | 0.45 (0.04) | |  |
|  | *Staphylococcus* |  |  |  |  |  |  |  |  |  |  |  |  |  | 0.45 (0.04) | |  |
|  | *Streptococcus* |  |  |  |  |  |  |  |  |  |  |  |  |  | 0.5 (0.02) | 0.45 (0.04) |  |
|  | *Tetragenococcus* |  |  |  |  |  |  |  |  |  |  |  |  |  | 0.5 (0.02) | |  |
|  | *Blautia* | -0.58 (0.004) | | -0.45 (0.03) | | | |  |  |  |  |  |  |  |  | |  |
|  | *Lachnospiraceae* (family) | -0.81 (<0.001) | -0.73 (<0.001) | 0.56 (0.006) | -0.57 (0.005) | -0.39 (0.06) | -0.54 (0.008) |  |  |  |  |  |  |  |  |  |  |
|  | *Lachnospiraceae* ND3007 (family) | 0.41 (0.05) | |  | | | |  |  |  |  |  |  |  |  |  |  |
|  | *Lachnospiraceae* UCG-004 (family) | -0.79 (< 0.001) | -0.66 (< 0.001) | -0.61 (0.002) | | -0.59 (0.003) | |  |  |  |  |  |  |  |  |  |  |
|  | *Oribacterium* | -0.79 (< 0.001) | | -0.57 (0.002) | | | |  |  |  |  |  |  |  |  |  |  |
|  | *Coprococcus* |  | |  | | | | 0.46 (0.03) | |  |  |  |  |  |  |  |  |
|  | *Erysipelotricheaceae* UCG-003 |  |  |  |  |  |  | -0.44 (0.03) | -0.44 (0.03) |  |  |  |  |  |  |  |  |
|  | *Holdemania* |  |  |  |  |  |  | -0.48 (0.02) | |  |  |  |  |  |  |  |  |
|  | *Megamonas* |  |  |  |  |  |  |  | |  |  |  |  |  | 0.43 (0.04) | |  |
|  | *Dialister* |  |  |  |  |  |  |  |  | 0.54 (0.01) | | | -0.46 (0.03) | |  | |  |
| Proteobacteria | *Pelagibacter* |  |  |  |  |  |  |  |  |  | | | | |  |  | -0.48 (0.02) |
|  | *Neisseria* |  |  |  |  |  |  | -0.43 (0.04) | |  |  |  |  |  |  |  | -0.48 (0.04) |
|  | *Parasutterella* |  |  |  |  |  |  | 0.42 (0.05) | |  |  |  |  |  |  |  |  |
|  | *Sutterella* |  |  |  |  |  |  | -0.47 (0.02) | -0.47 (0.02) | 0.54 (0.008) | | | 0.54 (0.008) | |  |  |  |
|  | *Oxalobacter* |  |  |  |  |  |  |  | | 0.48 (0.02) | | | | |  |  |  |
|  | *Desulfovibrio* |  |  |  |  |  |  |  |  | 0.54 (0.008) | 0.54 (0.008) | 0.54 (0.008) | 0.48 (0.02) | 0.48 (0.02) |  |  |  |
|  | *Bilophila* |  |  |  |  |  |  |  |  | 0.46 (0.03) | | | 0.48 (0.02) | |  |  |  |
|  | *Helicobacter* |  |  |  |  |  |  |  |  | 0.54 (0.008) | | | 0.54 (0.008) | |  |  |  |
|  | *Citrobacter* |  |  |  |  |  |  | 0.42 (0.05) | |  | | | | |  |  |  |
|  | *Pseudoalteromonas* |  |  |  |  |  |  |  | | 0.54 (0.008) | | | | |  |  |  |
|  | *Succinivibrio* |  |  |  |  |  |  |  |  | 0.54 (0.008) | | | 0.54 (0.008) | |  |  |  |

Correlation coefficient is shown next to P value in parentheses.
